# Modern physiological approach to inappropriate ICD shocks due to atrial fibrillation with very fast ventricular response. A case report

**DOI:** 10.1186/s12877-024-04862-0

**Published:** 2024-03-12

**Authors:** Catalin Pestrea, Roxana Enache, Ecaterina Cicala, Radu Vatasescu

**Affiliations:** 1 Interventional Cardiology Unit, Brasov County Clinical Emergency Hospital, 500326 Brasov, Romania; 2 Department of Medical and Surgical Specialties, Faculty of Medicine, “Transilvania” University of Brasov, 500019 Brasov, Romania; 3 Faculty of Medicine, Carol Davila University of Medicine and Pharmacy, 050474 Bucharest, Romania; 4 Electrophysiology and Cardiac Pacing Lab, Clinical Emergency Hospital, 014461 Bucharest, Romania

**Keywords:** DF-1 defibrillator, Inappropriate shocks, Left bundle branch area pacing, Atrioventricular node ablation

## Abstract

**Background:**

Fast-conducting atrial fibrillation misinterpreted as ventricular tachycardia is the leading cause for inappropriate shocks in patients with implantable cardiac defibrillators (ICD). These inappropriate shocks are associated with significant morbidity and mortality and cause great discomfort and stress.

**Case presentation:**

We report the case of a patient with ischemic cardiomyopathy, permanent atrial fibrillation, and a single-chamber DF-1 ICD implanted for the primary prevention of sudden cardiac death, who presented for multiple inappropriate internal shocks due to very fast-conducting atrial fibrillation, which was mislabeled as ventricular fibrillation by the ICD. Since the patient was under maximal atrioventricular nodal blocking medical therapy (beta-blockers and digitalis) and we didn`t find any reversible causes for the heart rate acceleration, we opted for rate control with atrioventricular node ablation. To counteract the risk of pacing-induced cardiomyopathy in this patient who would become totally pacemaker-dependent, we successfully performed left bundle branch area pacing. Because the patient`s ICD had a DF-1 connection and the battery had a long life remaining, we connected the physiological pacing lead to the IS-1 sense-pace port of the ICD. The 6-month follow-up showed an improvement in left ventricular function with no more inappropriate shocks.

**Conclusions:**

Left bundle branch area pacing and atrioventricular node ablation in patients with an implantable single-chamber DF-1 defibrillator and fast-conducting permanent atrial fibrillation is a cost-efficient and very effective method to prevent and treat inappropriate shocks, avoiding the use of an additional dual-chamber or CRT-D device.

## Background

Inappropriate shocks in patients with implantable cardiac defibrillators (ICD) are associated with significant morbidity and mortality and generate great discomfort and stress [1]. The leading cause is fast-conducting atrial fibrillation misinterpreted as ventricular tachycardia. We present the case of a patient with multiple inappropriate shocks due to fast-conducting atrial fibrillation (AF) who was effectively treated with atrioventricular node (AVN) ablation and left bundle branch area pacing (LBBAP) using the existing single-chamber ICD.

## Case presentation

**Fig. 1:**
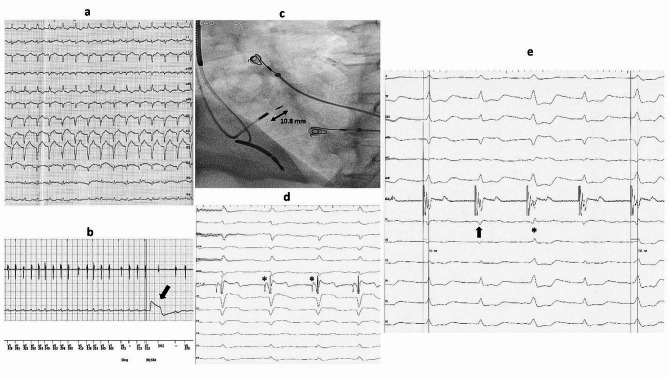
**(a)** Presenting 12-lead ECG (at 25 mm/sec sweep speed) with rapid conducting atypical atrial flutter/AF **(b)** Episode stored in the ICD`s memory showing interpretation of fast, irregular AF as ventricular fibrillation and delivery of an internal shock (black arrow) **(c)** Depth of penetration of the lead into the interventricular septum (from lead tip to the proximal electrode − 10.8 mm) **(d)** 12-lead ECG (at 50 mm/sec sweep speed) and intracardiac electrogram recorded from the lead tip depicting a large proximal LBB potential (asterisk) **(e)** Pacing at that site with decremental amplitude revealed a transition from non-selective LBB pacing (with an LVAT of 95 ms– black arrow) to left septal myocardial capture (wider QRS and an LVAT of 111 ms - asterisk). ECG– electrocardiogram; AF– atrial fibrillation; LBB– left bundle branch; LVAT– left ventricular activation time

**Fig. 2:**
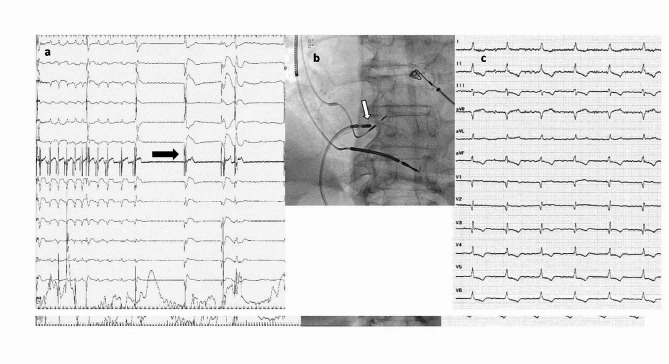
**(a)** 12-lead ECG (at 25 mm/sec sweep speed) and intracardiac electrogram recorded from the lead tip showing the moment of AVN ablation (black arrow) **(b)** Posteroanterior X-ray image showing the successful ablation site at a safe distance from the LBB pacing lead tip (white arrow) **(c)** Final 12-lead ECG (at 25 mm/sec sweep speed) depicting regular and narrow QRS complexes. ECG– electrocardiogram; AVN– atrioventricular node; LBB– left bundle branch

A 65-year-old male with ischemic cardiomyopathy, permanent AF (diagnosed for five years), and a single-chamber DF-1 ICD for primary prevention was brought to the emergency department for multiple internal electrical shocks. He had his first device implanted 12 years ago and subsequently underwent two box changes, with the last being two years prior to the current presentation. His presenting ECG showed atypical atrial flutter/AF with a fast ventricular rate of 180 bpm (Fig. 1a). Device interrogation revealed a total of 51 shock therapies, all for rapid AF, which were mislabeled as ventricular fibrillation (Fig. 1b). No clinical signs or symptoms of decompensated heart failure were obvious on physical examination. The laboratory findings were unremarkable, including normal blood count and thyroid function. The echocardiography showed an enlarged left ventricle with a severely depressed ejection fraction (30%), mild valvular regurgitations, and a dilated left atrium with a diameter of 50 mm and a volume of 105 ml. The ICD had a ventricular tachycardia detection zone programmed at 170 bpm and a ventricular fibrillation detection zone programmed at 200 bpm with an initial detection duration of 3 s. Since most of the shocks were delivered for rates well above 200 bpm, we considered that reprogramming the device would be futile. So, because the patient was on optimal heart failure therapy (including an angiotensin receptor/neprilysin inhibitor, mineralocorticoid antagonist, and sodium-glucose co-transporter-2) and maximum AVN blocking agents (10 mg of Bisoprolol and 0.25 mg of Digoxin five days per week), a decision was made to control the heart rate with a physiological pace and ablate strategy. Successful LBBAP was first performed (Fig. 1c) using a Medtronic C315 His catheter (Medtronic, Minneapolis, MN, USA) and a Medtronic SelectSecure 3830 lead (Medtronic, Minneapolis, MN, USA), with the identification of a large proximal left bundle branch potential (Fig. 1d) and non-selective capture resulting in a paced QRS complex of 110 ms duration (Figs. 1e and 2c). The capture threshold for the left bundle branch was 0.7 V at 0.4 ms and the sensing values were above 12 mV. The lead was connected to the existing defibrillator`s IS-1 sense-pace port and the defibrillator coils to the corresponding high-energy ports. During the same procedure, successful ablation of the AVN was performed (Fig. 2a,b). The device was programmed with a single ventricular fibrillation detection zone at 200 bpm with an initial detection duration of 5 s. The patient was discharged the next day. The 6-month follow-up showed constant pacing and sensing thresholds, no inappropriate shocks, and an improvement in ejection fraction to 40%.

## Discussion

In the permanent form of AF, besides chronic anticoagulation, the only option for treatment is rate control. Unfortunately, in some patients, especially those of a younger age who have very good AVN conduction, adequate rate control with beta-blockers and/or digitalis is difficult to achieve. In active patients, during physical exertion, the heart rate can significantly increase up to 200 bpm, despite optimal drug doses [2].

At these high rates, most single-chamber ICDs cannot perform adequate discrimination between supraventricular and ventricular arrhythmias, since the heart rate usually falls into the ventricular fibrillation programmed window. Additionally, faster heart rates contribute to the decrease in ejection fraction and maintenance of heart failure symptoms despite adequate treatment in these patients.

A definitive solution to control the heart rate in permanent AF patients is AVN ablation [3]. Unfortunately, this therapy renders the patient pacemaker-dependent. Given that, an important percentage of patients wearing ICDs have a reduced ejection fraction, conventional right ventricular pacing is a suboptimal choice since long-term, high ventricular pacing burden is associated with a significant further decrease in left ventricular performance [4].

Current guidelines recommend for patients with a baseline reduced ejection fraction and an expected high-pacing burden, either biventricular pacing or physiological pacing in the form of His bundle pacing (HBP) or LBBAP [5].

The option of biventricular pacing with defibrillator support requires the implantation of a CRT-D device, with significant procedural costs. On the other hand, if one chooses conduction system pacing, there are several options available, including the use of the IS-1 port of a DF-1 defibrillator as we did in our case. Our patient already had an ICD implanted recently with a very good battery life, so changing the device would have been a waste of resources. We opted for LBBAP instead of HBP for several reasons. In HBP there is a risk for capture threshold increase over time with faster battery drainage [6]. Also, in HBP, ventricular sensing is usually low and there is the potentially fatal risk of ventricular undersensing of fibrillatory waves. Thirdly, the risk of tissue damage at the lead tip during AVN ablation is minimal, since the distance from the lead to the ablation site offers a good safety margin [7].

One previous study including patients with persistent AF and ICDs showed that, in the group with AVN ablation and conduction system pacing, there were no more inappropriate shocks and there was a significant improvement in left ventricular function, compared to the optimal medical therapy group. An important observation is that, in the former group, the conduction system pacing lead was connected to the atrial port of a dual-chamber ICD or the left ventricular port of a CRT-D device [8].

Small previous studies investigating this strategy of placing the LBBAP lead in the IS-1 port of a DF-1 defibrillator have shown safety and adequate sensing during defibrillation threshold testing [9]. In our patient, because the sensing values were optimal, we decided not to perform a defibrillation test.

As previously shown in feasibility studies, the pacing and sensing parameters with LBBAP are usually stable, as was the case of our patient at 6 months follow-up [10]. From the clinical perspective, the added benefit of definitive rate control and physiological depolarization led to an improvement in left ventricular function with no more inappropriate shocks recorded.

## Conclusions

LBBAP and AVN ablation in patients with an implantable single-chamber DF-1 defibrillator and fast-conducting permanent AF is a cost-efficient and very effective method to prevent and treat inappropriate shocks, avoiding the use of an additional dual-chamber or CRT-D device.

## Data Availability

Data sharing is not applicable to this article as no datasets were generated or analyzed during the current study.

## Abbreviations

ICD: Implantable Cardiac Defibrillator
AF: Atrial Fibrillation
LBBAP: Left Bundle Branch Area Pacing
HBP: His Bundle Pacing
AVN: Atrioventricular Node
ECG: Electrocardiogram
CRT-D: Triple-Chamber Defibrillator
